# Editorial: Sepsis and COVID-19: Cross-Talk in Signaling Pathways and in Therapeutic Perspectives

**DOI:** 10.3389/fmed.2022.917792

**Published:** 2022-05-30

**Authors:** Reinaldo Salomão, Fernando Queiróz Cunha, Felipe Dal-Pizzol

**Affiliations:** ^1^Infectious Disease Department, Federal University of São Paulo, São Paulo, Brazil; ^2^Center of Research in Inflammatory Diseases (CRID), Department of Pharmacology, Ribeirão Preto Medical School, University of São Paulo, São Paulo, Brazil; ^3^Laboratory of Experimental Pathophysiology, Graduate Program in Health Sciences, Universidade do Extremo Sul Catarinense, Criciúma, Brazil

**Keywords:** sepsis, COVID-19, predictor factors, pathogenesis, epigenetic

Sepsis, as a manifestation of several endemic and epidemic diseases, has had a profound impact on humankind's history. In the last decades, sepsis remained a major cause of morbidity and mortality worldwide. In December 2019, the city of Wuhan, in China, became the center of an outbreak of pneumonia of unknown cause, rapidly identified as triggered by a new coronavirus, the SARS-CoV-2 (from “severe acute respiratory syndrome coronavirus 2”). The disease was characterized as COVID-19 (an acronym for “coronavirus disease”) by the World Health Organization (WHO February 11, 2020, and in just a month, on March 11, it was declared a global pandemic (WHO Director-General's opening remarks at the media briefing on COVID19 -March 2020). As of 8 April 2022, there have been circa 500 million confirmed cases of COVID-19, including over 6 million deaths reported to (1).

Patients infected with SARS-CoV-2 and progressing to critical COVID-19 illness are unequivocally presenting sepsis. However, there are important differences in the clinical trajectories and underlying mechanisms driving to critical disease between a COVID-19 patient and a regular septic patient. COVID-19 is a disease and sepsis is a syndrome. COVID-19 patients deteriorating to sepsis and septic shock show a typical clinical course, the medium time from symptoms' onset to dyspnea ranging from 5 to 8 days, the median time to acute respiratory distress syndrome (ARDS) from 8 to 12 days, and the median time to ICU admission ranging from 10 to 12 days. This is in frank contrast with the unpredictable or heterogeneous timeframe of events in a regular septic patient and has been a clue for a better understanding of the pathophysiological events as well as to achieve more success in adjunctive therapy strategies.

Although present important differences, the pathogenesis of sepsis and COVID-19 converges to a pivotal role in host systemic inflammatory response. Cytokines storm, procoagulant state, Toll-Like Receptor (TLR) signaling, Pathogen-associated Molecular Patterns (PAMPs) and Damage-Associated Molecular Patterns (DAMPs), neutrophil extracellular traps (NETs), inflammasome, changes in lipids profile are involved in both diseases. Thus, the sepsis literature quickly became COVID literature as well, and this overlap highlights our need to better phenotyping regular sepsis.

This Research Topic covered a wide scope of sepsis and COVID-19 interfaces, including clinical profiles and sequelae, translational research evaluating biomarkers and predictive outcomes, and mechanisms underlying the dysregulated inflammatory response.

Abumayyaleh et al. developed a score aiming to help clinicians in identifying high-risk COVID-19 patients progressing to sepsis while Alencar et al. tested the performance of NEWS, qSOFA, and SIRS scores for assessing mortality, early bacterial infection, and admission to ICU in covid-19 patients admitted in the emergency department. Zhang et al. reported on clinical similarities and differences between acute lung injury (ALI) in COVID-19 and non-COVID-19 patients in the intensive care unit (ICU), and Prestes et al. showed that long term lung dysfunction is common in patients with severe COVID-19 and impacts negatively on activities of daily living

Pathogenetic changes are related to clinical outcomes or envisaged as oriented target therapy. Microangiopathy and thrombosis coupled with dysfunctional local inflammatory response are the basis for organ dysfunction in COVID-19. Here, Maldonado et al. observed increased concentrations of thrombomodulin, angiopoietin-2, human vascular endothelial growth factor, and human hepatocyte growth factor and a decrease in human tissue inhibitor of metalloproteinases-2 in COVID-19 patients, and demonstrated that early endothelial and angiogenic biomarkers could predict mortality in patients with COVID-19.

A dysregulated immune response with concomitant pro-inflammatory and immune suppressive responses is one of the fundamental changes observed in sepsis. Alon et al. bring evidence that CD45/TCR intracellular signaling is downregulated in peripheral blood mononuclear cells from COVID-19 patients and demonstrate that C24D, an immunomodulatory peptide, rescued CD45 signaling. On the other hand, the robust data on dysfunctional inflammatory response and the pivotal role of “NETosis”, the process of neutrophil release of their extracellular traps, is discussed in a comprehensive review by Keane et al.

In an interesting approach, Bouwman et al. addressed signal transduction pathway activities in whole blood samples from patients with sepsis and observed increased activity in androgen (AR) receptor and TGFβ pathways. AR showed a good performance for diagnosing and predicting sepsis' outcomes.

There is increasing evidence that immune and metabolic response during an infectious process is under epigenetic control. Epigenetic changes play an important role in regulating DNA processes, such as transcription, repair, and replication. Falcão-Holanda et al. review the epigenetic changes reported in experimental and clinical studies and discuss their role in the pathogenesis of sepsis and as a potential target for adjunctive therapy.

The rapid development of vaccines against COVID-19, based on different platforms, from the traditional inactivated viruses to the new mRNA technology, was an unprecedented scientific achievement and a central approach to pandemic control. As of 5 April 2022, over 11 billion vaccine doses have been administered in the world (WHO, 2022). One concern with this massive immunization is the possible side effects, among others, related to thromboembolism, myocarditis/pericarditis, and neuropathies. Here, Di Mauro et al. focused on acute vertigo syndrome, which, as they pointed out, could represent an overlap between ear/labyrinth and nervous system disorders following COVID-19 immunization.

The pathogenesis-oriented target therapy also converges sepsis and COVID-19. In COVID-19, as was formerly the case with sepsis, a great enthusiasm was observed with multiple inflammation-based targets for intervention, the IL-6 inhibitors as an illustrating example. On the other hand, targeting the immunosuppressive state with IL-7 has undergone clinical trials as well. Again, deciphering the host-protective defense from the harmful response and identifying patients which would benefit from one or other approach are a pivotal research challenge.

We are in debt to the authors of the Research Topic for the excellence of their contributions and hope that the topic will contribute with our increasing knowledge about sepsis and COVID-19.

## Author Contributions

All authors listed have made a substantial, direct, and intellectual contribution to the work and approved it for publication.

## Conflict of Interest

The authors declare that the research was conducted in the absence of any commercial or financial relationships that could be construed as a potential conflict of interest.

## Publisher's Note

All claims expressed in this article are solely those of the authors and do not necessarily represent those of their affiliated organizations, or those of the publisher, the editors and the reviewers. Any product that may be evaluated in this article, or claim that may be made by its manufacturer, is not guaranteed or endorsed by the publisher.

## References

[1] World Health Organization (2022). WHO Coronavirus Disease (COVID-19) Dashboard. (2022). Available online at: https://covid19.who.int (accessed April 9, 2022).

